# Supplementary material for: Kupffer cell and recruited macrophage heterogeneity orchestrate granuloma maturation and hepatic immunity in visceral leishmaniasis
Source: Nat Commun. 2025 Apr 1;16:3125. doi: 10.1038/s41467-025-58360-x (PMC11961706; doi:10.1038/s41467-025-58360-x)
Supplement: Supplementary file 1 — Supplementary Information [file 41467_2025_58360_MOESM1_ESM.pdf]

Title: Kupffer cell and recruited macrophage heterogeneity orchestrate granuloma maturation and hepatic immunity in visceral leishmaniasis

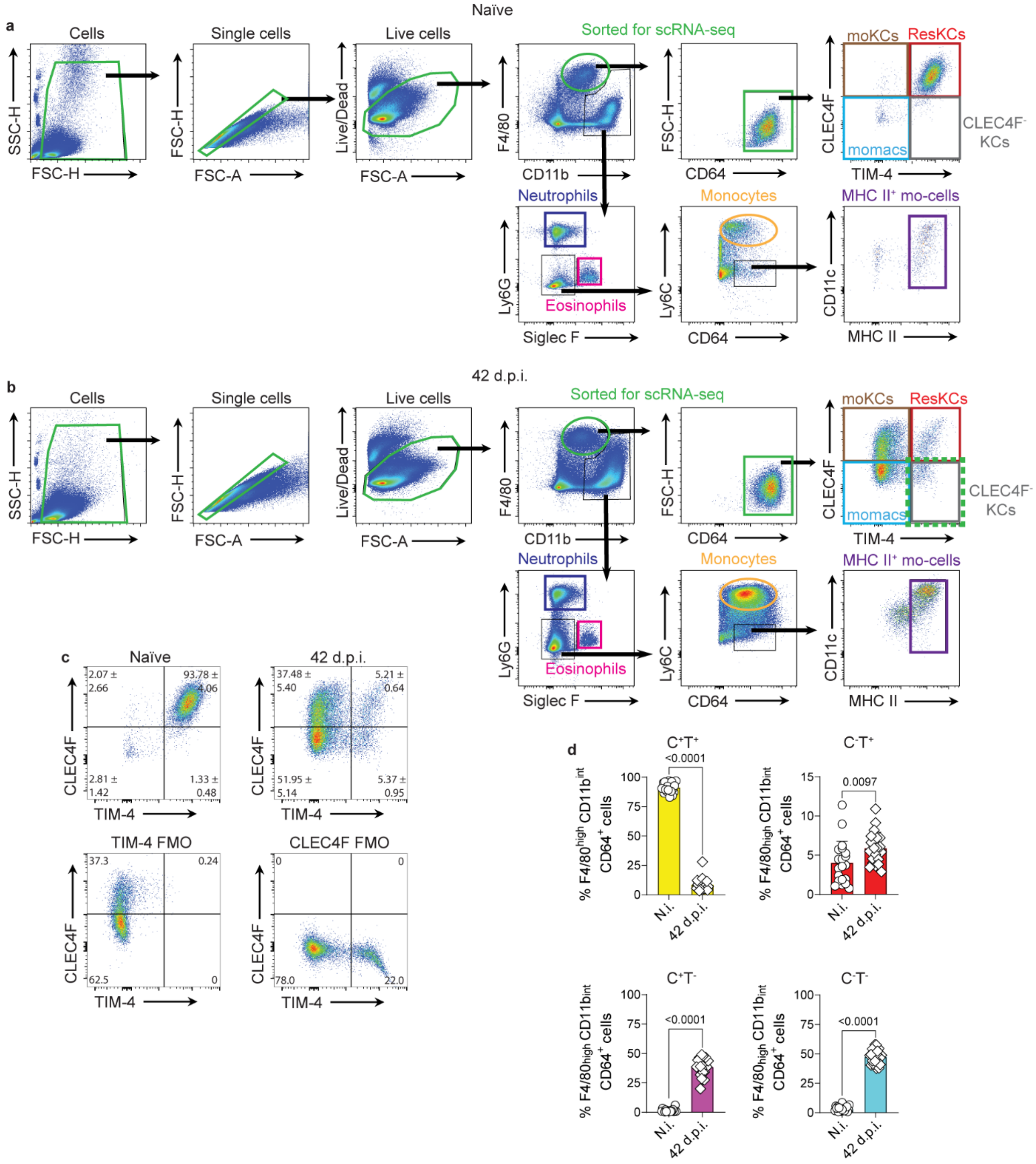

Supplementary Fig. 1: Myeloid cells gating strategy and macrophage heterogeneity at 42 days post-infection. a-b, Dot plots showing the gating strategy used to identify resKCs ($F4/80^{hi}CD11b^{int}CD64^{+}CLEC4F^{+}TIM\text{-}4^{+}$), neutrophils ($CD11b^{+}Ly6G^{+}SiglecF^{-}$), eosinophils ($CD11b^{+}Ly6G^{-}SiglecF^{+}$), monocytes ($CD11b^{+}Ly6G^{-}SiglecF^{-}Ly6C^{+}CD64^{+}$), and monocyte-derived cells ($CD11b^{+}Ly6G^{-}SiglecF^{-}Ly6C^{+}CD64^{+}MHCII^{+}$) in live, single cells isolated from naïve **(a)** and 42-day infected livers **(b)**. Green gates designate the macrophages used for the first scRNA-seq, and green gates plus dashed green gate represent the $CLEC4F^{-}$ cells used in the second scRNA-seq. **c**, Representative FACS plots showing CLEC4F and TIM-4 expression gated on $F4/80^{hi}CD11b^{int}CD64^{+}$ macrophages in naïve and 42-day infected mice, along with fluorescence minus one (FMO) staining for CLEC4F and TIM-4. FMO controls consist of mixed naïve and infected cells. Numbers indicate mean ± SD percentage of cells in the gate. **d**, Frequency of macrophage subsets in naïve and 42-day infected mice by flow cytometry. Data pooled from 5 independent experiments (n=20). Values represent the mean ± SD. For data that passed the normality test, *P* values were obtained using a two-tailed unpaired *t* test. For data that did not pass the normality test, a two-tailed Mann-Whitney test was used. Source data are provided as a Source Data file.

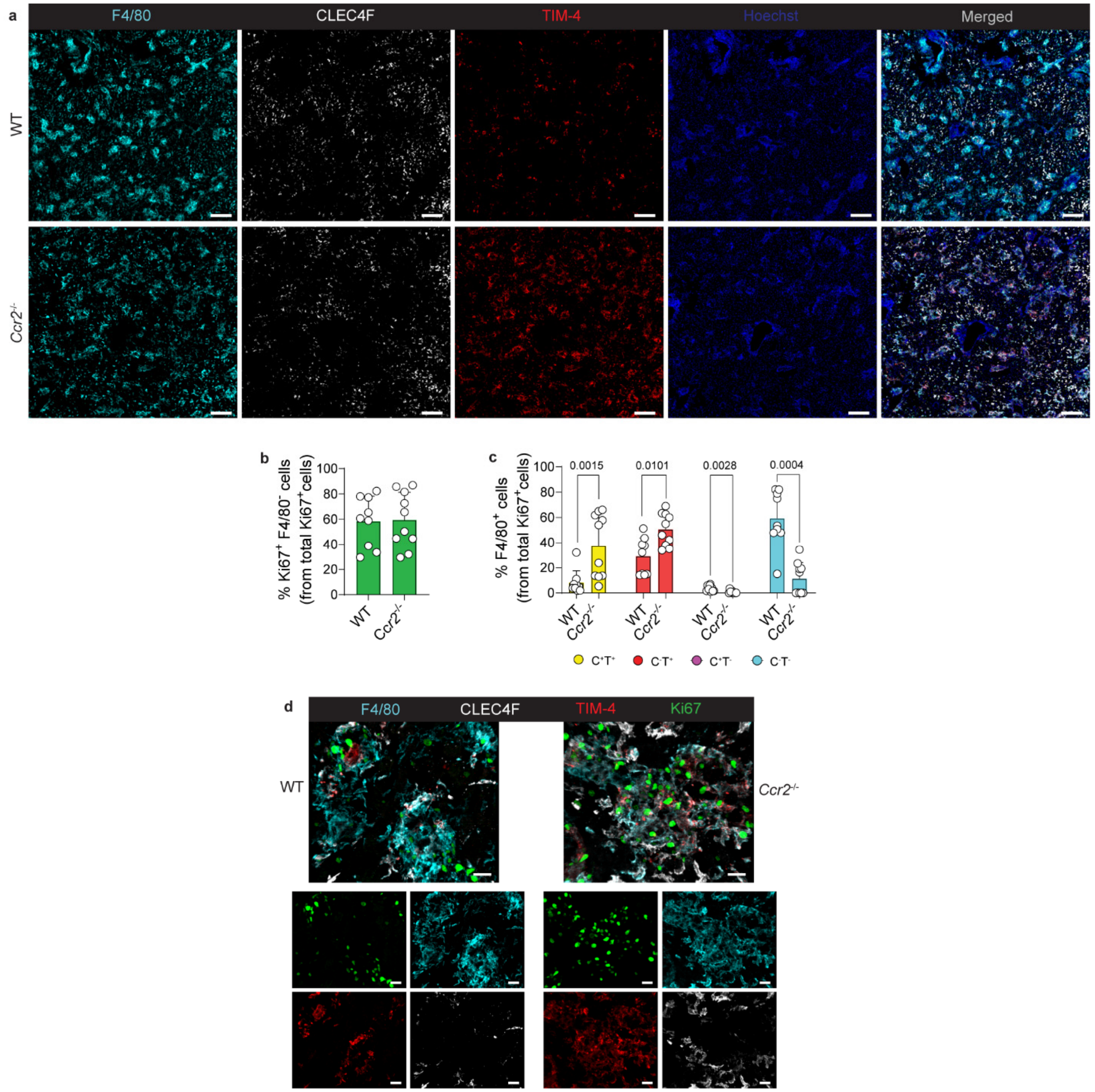

Supplementary Fig. 2: Cell proliferation in WT and *Ccr2*$^{-/-}$ infected livers. a, Original immunofluorescence images from rendered images in **Fig**.**2i**, showing WT and *Ccr2*$^{-/-}$ livers at 42 d.p.i., and F4/80 (cyan), CLEC4F (white), TIM-4 (red), and Hoechst (blue). Scale bars, 200 μm. **b**, Frequency of Ki-67^{+}F4/80^{-} proliferating cells as a percentage of all Ki-67^{+} cells in infected WT and *Ccr2*$^{-/-}$ mice at 42 d.p.i. **c**, Frequency of Ki-67^{+}F4/80^{+} macrophages as a percentage of all F4/80^{+} cells in WT and *Ccr2*$^{-/-}$ mice at 42 d.p.i. Data pooled from 2 independent experiments (n=9 for WT

and n=10 for *Ccr2*$^{-/-}$). **d**, Representative images of granulomas in WT and *Ccr2*$^{-/-}$ mice at 42 d.p.i., showing F4/80 (cyan), CLEC4F (white), TIM-4 (red), and Ki-67^{+} proliferating cells (green). Scale bars, 20 µm. Data pooled from 2 independent experiments (n=9 for WT and n=10 for *Ccr2*$^{-/-}$). Values in **b-c** represent the mean ± SD. *P* values were obtained using a two-tailed Mann-Whitney test. Source data are provided as a Source Data file.

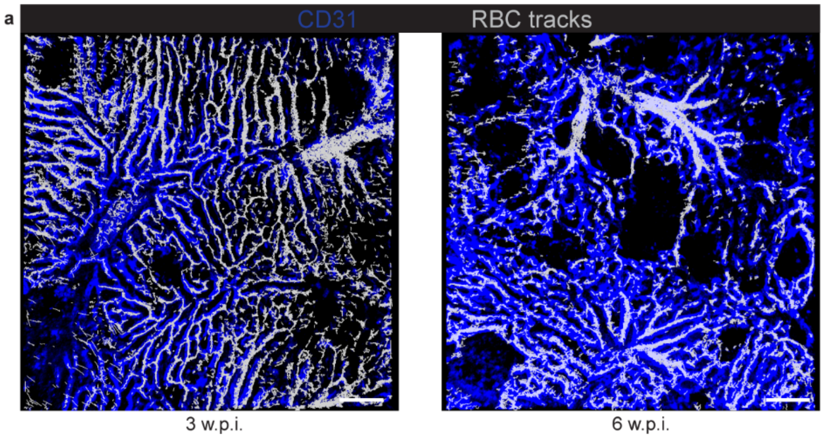

Supplementary Fig. 3: Red blood cells tracks during VL. a, Representative images showing the sinusoids (blue) and RBCs tracks (gray) in 3- and 6-week infected mice. Scale bars, 100 µm. Data from 2 independent experiments (n=2).

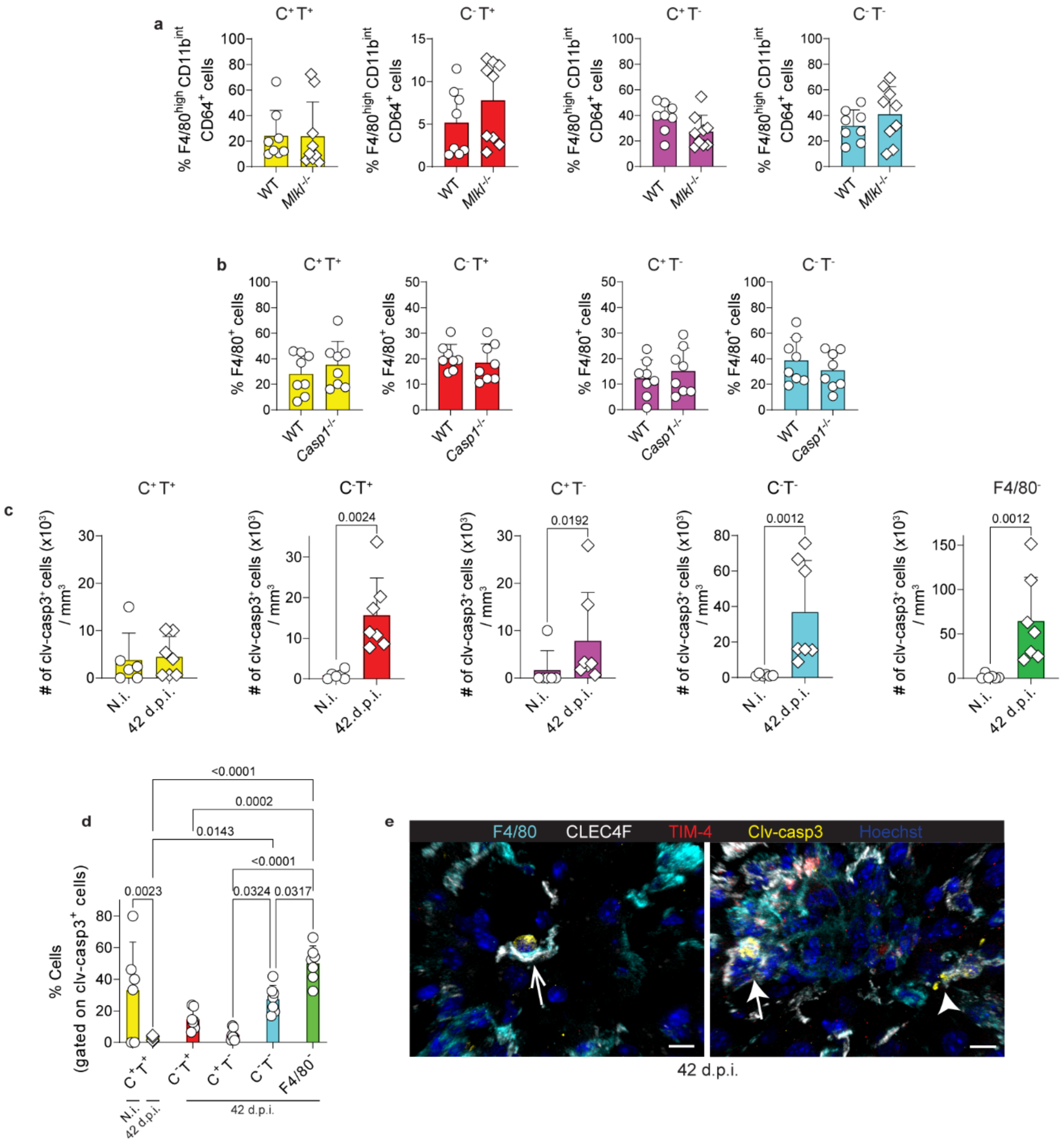

Supplementary Fig. 4: Cell death pathways in macrophages during late-stage VL. a, Frequency of cells in F4/80^{hi}CD11b^{int}CD64^{+} subsets in 42 d.p.i. WT and *Mlkl*$^{-/-}$ mice, by flow cytometry. Data pooled from 2 independent experiments (n=8 for WT and n=9 for *Mlkl*$^{-/-}$). **b**, Frequency of F4/80^{+} subsets in 42 d.p.i. WT and *Casp1*$^{-/-}$ mice, quantified from immunofluorescence images (n=8). **c**, Number of cleaved caspase 3^{+} cells in each F4/80^{+} subset and in F4/80^{-} cells, quantified from immunofluorescence images in naïve and 42-day infected WT

mice. Data pooled from 2 independent experiments (n=6 for naïve and n=7 for 42 d.p.i.). **d**, Frequency of each subset of F4/80$^+$ and F4/80$^-$ cells, gated on cleaved caspase 3$^+$ cells, in naïve and 42-day infected WT mice. Data pooled from 2 independent experiments (n=6 for naïve and n=7 for 42 d.p.i.). **e**, Representative immunofluorescence images showing apoptotic CLEC4F$^+$TIM-4$^-$moKC (thin arrow), apoptotic CLEC4F$^-$TIM-4$^+$KC (arrow), and apoptotic CLEC4F$^+$TIM-4$^+$resKC (arrowhead) in 42-day infected mice. Scale bars, 8 and 10 μm, respectively. Values in **a-d** represent mean ± SD. In **a-c,** for data that passed the normality test, statistical analysis was performed using a two-tailed unpaired *t* test. For data that did not pass the normality test, statistical analysis was performed using a two-tailed Mann-Whitney test. In **d**, *P* values were obtained using ordinary one-way ANOVA with Sidak's multiple comparisons test. Source data are provided as a Source Data file.

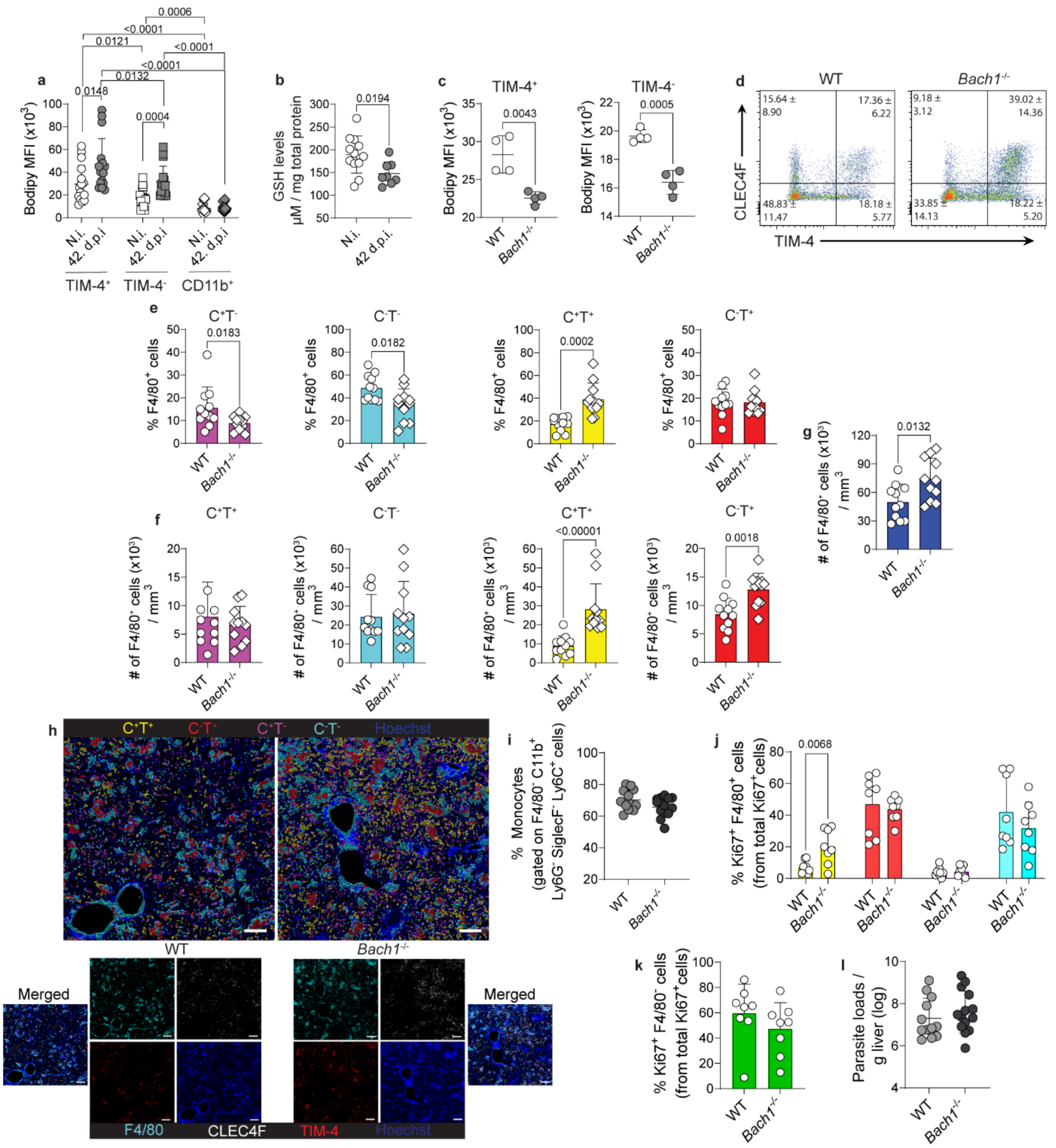

Supplementary Fig. 5: BACH1 regulation of resKC proliferation and macrophage lipid peroxidation. **a**, Lipid peroxidation in TIM-4$^{+/-}$ cells (gated on F4/80^{hi}CD11b^{int}CD64^{+} macrophages) and in CD11b^{+} cells (excluding F4/80^{hi} macrophages) in naïve and 42 d.p.i. mice, by flow cytometry. Data pooled from 4 independent experiments (n=16). **b**, Intracellular GSH levels measured in whole tissue homogenates from naïve and 42 d.p.i. mice. Data pooled from 3

independent experiments for naïve and 2 independent experiments for 42 d.p.i. (n=12 for naïve and n=8 for 42 d.p.i.). **c**, Lipid peroxidation in TIM-4$^+$ and TIM-4$^-$ cells from WT and *Bach1*$^{-/-}$ mice at 42 d.p.i. Data representative of 2 independent experiments (n=4 each). **d**, Representative dot plots from immunofluorescence images showing the frequencies of macrophages based on CLEC4F and TIM-4 expression in WT and *Bach1*$^{-/-}$ at 42 d.p.i. Numbers indicate mean ± SD percentage of cells in the gate. **e-f** Frequency (**e**) and number (**f**) of F4/80$^+$ subsets in 42-day infected WT and *Bach1*$^{-/-}$ mice, quantified from immunofluorescence images. **g**, Number of F4/80$^+$ macrophages in WT and *Bach1*$^{-/-}$ mice at 42 d.p.i., quantified from immunofluorescence images. Data pooled from 3 independent experiments (n=11). **h**, Representative rendered images of WT and *Bach1*$^{-/-}$ 42-day infected livers showing CLEC4F$^+$TIM-4$^+$resKCs(yellow), CLEC4F$^-$TIM-4$^+$KCs(red), CLEC4F$^+$TIM-4$^-$moKCs(magenta) and CLEC4F$^-$TIM-4$^-$momacs(cyan), along with original immunofluorescence images showing F4/80(cyan), CLEC4F(white), TIM-4(red) and Hoechst(blue). Scale bars, 200 μm. **i**, Frequency of Ly6C^{hi} monocytes in infected livers of WT and *Bach1*$^{-/-}$ mice at 42 d.p.i. by flow cytometry. **j-k**, Frequency of Ki-67$^+$F4/80$^+$ **(j)** and Ki-67$^+$F4/80$^-$ cells **(k)** at 42 d.p.i. in WT and *Bach1*$^{-/-}$ mice, by confocal microscopy. **l**, Parasite loads in WT and *Bach1*$^{-/-}$ mice at 42 d.p.i. (data in **i** and **l** were pooled from 3 independent experiments, n=12 for WT, 14 for *Bach1*$^{-/-}$; data in **j** and **k** were pooled from 2 independent experiments, n=8). For **a-c**, **e-g**, and **i-l**, statistical analysis was performed using a two-tailed unpaired *t* test for data that passed the normality test. For data that did not pass the normality test, a two-tailed Mann-Whitney test was used. Values represent mean ± SD. Source data are provided as a Source Data file.

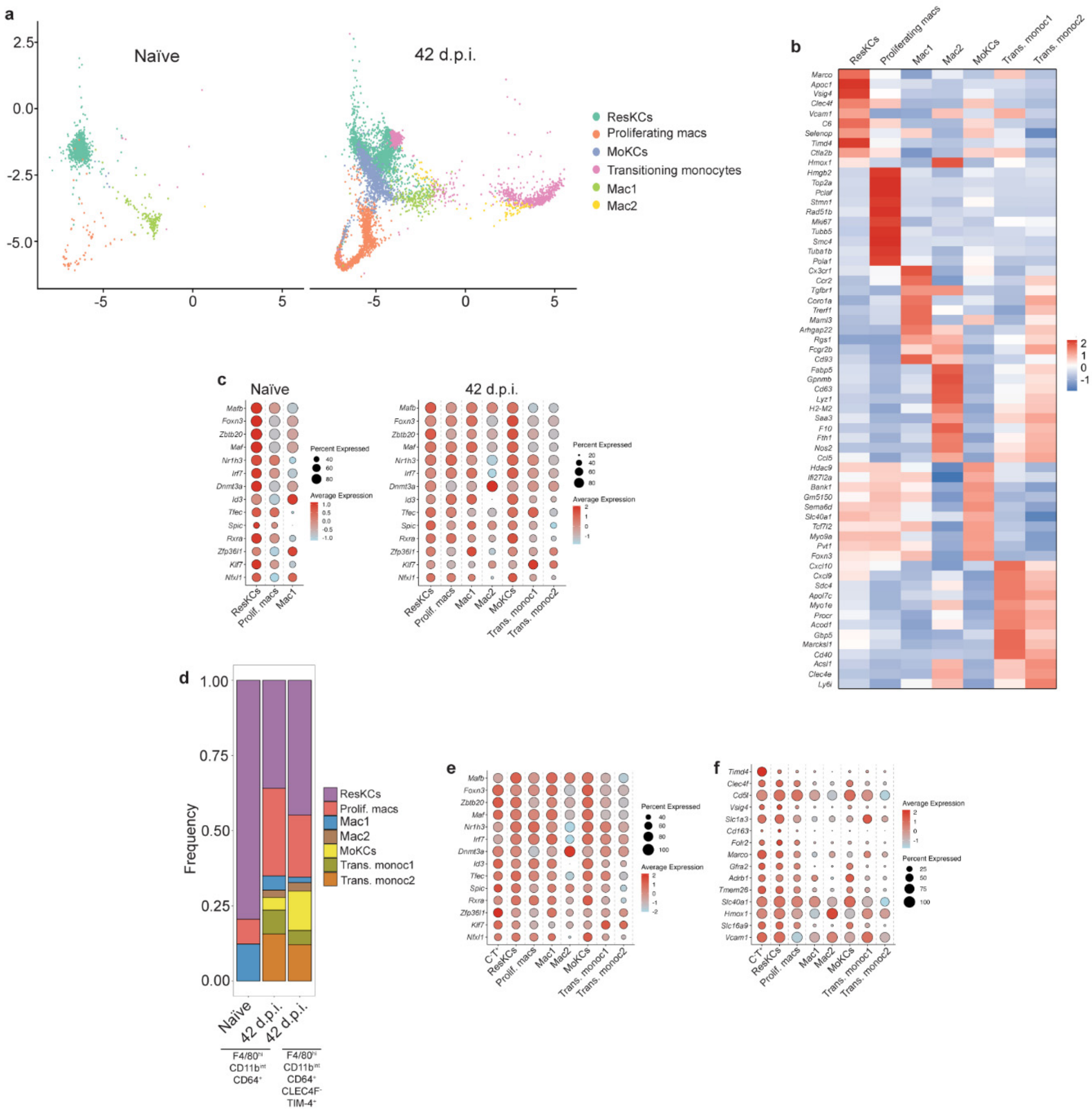

Supplementary Fig. 6: Clusters and differentially expressed genes identified by single-cell RNA sequencing in naïve and 42-day infected mice. a, UMAP plot of scRNA-seq data of sorted live, single, CD45.2$^+$F4/80$^+$CD11b^{int}CD64$^+$ cells from uninfected and 42 d.p.i. mice, showing 3 main clusters in uninfected and 6 clusters in infected mice, as defined by Remmerie *et al.*[14]. **b**, Heatmap showing the average expression of the top 10 differentially expressed genes that define the clusters from sorted live, single, CD45.2$^+$F4/80$^+$CD11b^{int}CD64$^+$ cells from uninfected and 42 d.p.i. mice. **c**, Average gene expression and corresponding cell percentage of KC-associated

transcription factors within each cluster. Data from 1,200 naïve cells and 6,152 cells from 42 d.p.i. mice, after QC filtering. **d**, The distribution of sorted CD45.2$^+$F4/80$^+$CD11b^{int}CD64$^+$CLEC4F$^-$TIM-4$^+$ cells in 42 d.p.i. mice overlaid with clusters from the scRNA-seq dataset from CD45.2$^+$F4/80$^+$CD11b^{int}CD64$^+$ sorted cells **e**, gene expression and corresponding cell percentage of KC-associated transcription factors **f**, KC signature genes as defined by Guilliams *et al.*[19] Data from 107 CD45.2$^+$F4/80$^+$CD11b^{int}CD64$^+$CLEC4F$^-$TIM-4$^+$ cells and 6,152 cells CD45.2$^+$F4/80$^+$CD11b^{int}CD64$^+$ from 42 d.p.i. mice, after QC filtering.

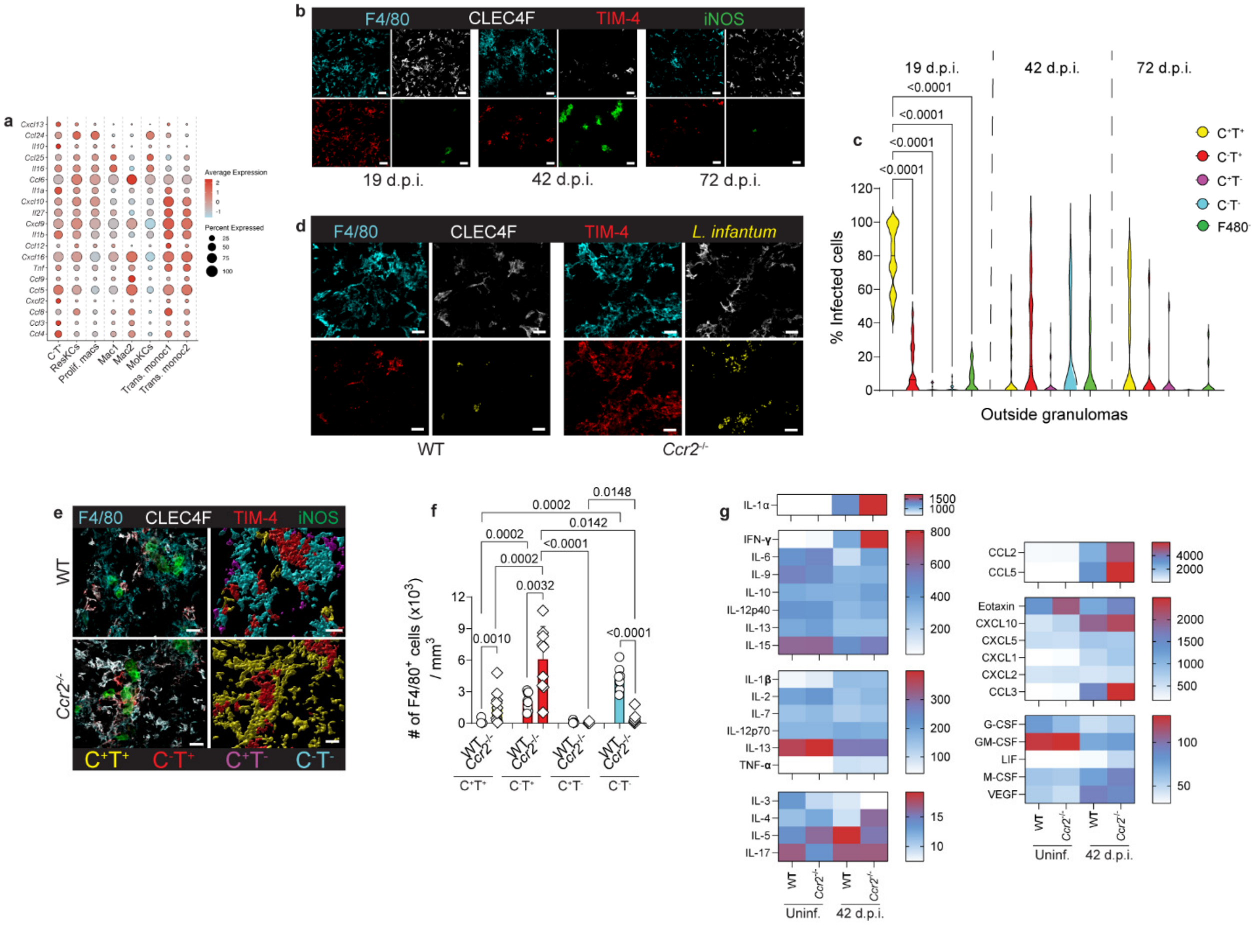

Supplementary Fig. 7: Activation and infection status of macrophage subsets during VL. **a**, Chemokines and cytokines expressed by CD45.2$^+$F4/80$^+$CD11b^{int}CD64$^+$CLEC4F$^-$TIM-4$^+$ sorted cells compared to CD45.2$^+$F4/80$^+$CD11b^{int}CD64$^+$ sorted cells at 42 d.p.i. **b**. Individual images from

Fig.6d showing F4/80 (cyan), CLEC4F (white), TIM-4 (red), and iNOS (green) in 19, 42-, and 72-day infected livers. Scale bars, 30μm. **c**, Frequency of infected cells identified outside F4/80$^+$ granulomas at 19 and 42 d.p.i., obtained from immunofluorescence images. Data pooled from 2 independent experiments for each time point and using 8 mice for 19- and 42 d.p.i., and 7 mice for 72 d.p.i. Frequencies were obtained from 2-3 regions containing granulomas from each liver (ROIs=23 for 19 d.p.i., ROIs=23 for 42 d.p.i., ROIs=12 for 72 d.p.i.). **d**, Individual images from merged images in **Fig.6n** showing F4/80 (cyan), CLEC4F (white), TIM-4 (red), and *L. infantum* (yellow) in WT and *Ccr2*$^{-/-}$ 42-day infected livers. Scale bars, 20μm. **e**, Representative and rendered images of F4/80$^+$ granulomas and iNOS expression in WT and *Ccr2*$^{-/-}$ 42-day infected mice, showing F4/80 (cyan), CLEC4F (white), TIM-4 (red), and iNOS (green) staining, and rendered F4/80$^+$ CLEC4F$^+$TIM-4$^+$resKCs(yellow), CLEC4F$^-$TIM-4$^+$KCs (red), CLEC4F$^+$TIM-4$^-$ moKCs (magenta), and CLEC4F$^-$TIM-4$^-$ (cyan) momacs. Scale bars, 30μm. **f**, iNOS expression by different F4/80$^+$ subsets in WT and *Ccr2*$^{-/-}$ mice at 42 d.p.i., quantified from immunofluorescence images. Data pooled from 2 independent experiments (n=8 for WT and n=9 for *Ccr2*$^{-/-}$). **g**, Cytokines and chemokines levels measured by Luminex in liver homogenates from WT and *Ccr2*$^{-/-}$ mice at 42 d.p.i. Data pooled from 2 independent experiments (n=9 for WT and n=8 for *Ccr2*$^{-/-}$). Values in **c, f** represent the mean ± SD. In **c**, *P* values were obtained using Kruskal-Wallis test with Dunn's multiple comparisons test. In **f**, for data that passed the normality test, *P* values were obtained using a two-tailed unpaired *t* test. For data that did not pass the normality test, *P* values were obtained using a two-tailed Mann-Whitney test. Source data are provided as a Source Data file.
