# Supplementary material for: Kupffer cell and recruited macrophage heterogeneity orchestrate granuloma maturation and hepatic immunity in visceral leishmaniasis
Source: Nat Commun. 2025 Apr 1;16:3125. doi: 10.1038/s41467-025-58360-x (PMC11961706; doi:10.1038/s41467-025-58360-x)
Supplement: Supplementary file 2 — Description of Additional Supplementary Files [file 41467_2025_58360_MOESM2_ESM.pdf]

Description of Additional Supplementary Files

Supplementary Data 1: List of differentially expressed genes in clusters identified by single-cell RNA sequencing in sorted CD45.2$^+$F4/80$^+$CD11b^{int}CD64$^+$ cells from uninfected and 42-day infected livers.

Supplementary Data 2: List of differentially expressed genes in clusters identified by single-cell RNA sequencing in sorted CD45.2$^+$F4/80$^+$CD11b^{int}CD64$^+$CLEC4F$^-$TIM-4$^+$ cells from 42-day infected livers.

Supplementary Movie 1: Small early-stage granulomas do not affect sinusoidal distribution. Rendered animation of a liver at 19 d.p.i., showing small Zsgreen$^+$ clusters (green) and intact sinusoids (magenta).

Supplementary Movie 2: Late-stage granulomas are located outside the sinusoids. Rendered animation of a liver at 42 d.p.i., depicting tdTomato (red), Zsgreen (green), and sinusoids (magenta). tdTomato$^+$ZsGreen$^+$KCs remain within the sinusoids, while tdTomato$^-$ZsGreen$^+$KCs clusters are observed outside the sinusoids.

Supplementary Movie 3: Displacement of the sinusoidal network by late-stage granulomas. Animation of a liver at 42d.p.i., showing tdTomato (red), Zsgreen (green) and sinusoids (magenta). Clusters of tdTomato$^-$ZsGreen$^+$KCs displace the surrounding sinusoids without changes in blood vessel diameter.

Supplementary Movies 4 and 5: RBCs tracking during early-stage *L. infantum* infection. Video showing an infected liver at 3w.p.i., depicting RBCs (red), F4/80 (green), and sinusoids (blue). Movie S5 is an animation version of movie S4.

Supplementary Movies 6 and 7: RBCs tracking during late-stage *L. infantum* infection. Video showing an infected liver at 6w.p.i, depicting RBCs (red), F4/80 (green), and sinusoids (blue). Movie S7 is an animation version of movie S6.
