# Supplementary material for: Kupffer cell and recruited macrophage heterogeneity orchestrate granuloma maturation and hepatic immunity in visceral leishmaniasis
Source: Nat Commun. 2025 Apr 1;16:3125. doi: 10.1038/s41467-025-58360-x (PMC11961706; doi:10.1038/s41467-025-58360-x)
Supplement: Supplementary file 12 — Reporting Summary [file 41467_2025_58360_MOESM12_ESM.pdf]

nature portfolio

Corresponding author(s): David L. Sacks

Last updated by author(s): Mar 3, 2025

Reporting Summary

Nature Portfolio wishes to improve the reproducibility of the work that we publish. This form provides structure for consistency and transparency in reporting. For further information on Nature Portfolio policies, see our Editorial Policies and the Editorial Policy Checklist.

Statistics

For all statistical analyses, confirm that the following items are present in the figure legend, table legend, main text, or Methods section.

n/a	Confirmed	
☐	☒	The exact sample size (*n*) for each experimental group/condition, given as a discrete number and unit of measurement
☐	☒	A statement on whether measurements were taken from distinct samples or whether the same sample was measured repeatedly
☐	☒	The statistical test(s) used AND whether they are one- or two-sided<br>*Only common tests should be described solely by name; describe more complex techniques in the Methods section.*
☒	☐	A description of all covariates tested
☐	☒	A description of any assumptions or corrections, such as tests of normality and adjustment for multiple comparisons
☐	☒	A full description of the statistical parameters including central tendency (e.g. means) or other basic estimates (e.g. regression coefficient) AND variation (e.g. standard deviation) or associated estimates of uncertainty (e.g. confidence intervals)
☐	☒	For null hypothesis testing, the test statistic (e.g. *F*, *t*, *r*) with confidence intervals, effect sizes, degrees of freedom and *P* value noted<br>*Give P values as exact values whenever suitable.*
☒	☐	For Bayesian analysis, information on the choice of priors and Markov chain Monte Carlo settings
☒	☐	For hierarchical and complex designs, identification of the appropriate level for tests and full reporting of outcomes
☒	☐	Estimates of effect sizes (e.g. Cohen's *d*, Pearson's *r*), indicating how they were calculated

Our web collection on statistics for biologists contains articles on many of the points above.

Software and code

Policy information about availability of computer code

Data collection	Flow cytometry: Cytek® Aurora cytometer and SpectroFlo (version 3.0.1 to 3.3.0).<br>Sorting: Cytek® Aurora CS and SpectroFlo (version 3.0.1 to 3.3.0).<br>Confocal microscopy: Leica SP8 WLL FLIM confocal microscope and LAS X Navigator software (version LAS X 3.5.5.19976).<br>Live imaging: Leica DIVE (Deep In Vivo Explorer) inverted microscope and LAS X Navigator software (version LAS X 3.5.5.19976).<br>scRNA-seq: Illumina HiSeqX Ten.
Data analysis	Flow cytometry: SpectroFlo® version 3.0.1 to 3.3.0 and FlowJo version 10.8.1 to 10.10.0.<br>Confocal microscopy: Imaris version 9.7.2 to 10.2.0 and FlowJo version 10.8.1 to 10.10.0.<br>For exporting channel intensity statistics from Imaris to FlowJo, source code is available at: https://doi.org/10.5281/zenodo.14970154<br>Statistical analysis: GraphPad Prism version 10.0.0 to 10.2.0.<br>scRNA-seq: RStudio version 2023.03.0.

For manuscripts utilizing custom algorithms or software that are central to the research but not yet described in published literature, software must be made available to editors and reviewers. We strongly encourage code deposition in a community repository (e.g. GitHub). See the Nature Portfolio guidelines for submitting code & software for further information.

Data

Policy information about availability of data

All manuscripts must include a data availability statement. This statement should provide the following information, where applicable:

- Accession codes, unique identifiers, or web links for publicly available datasets
- A description of any restrictions on data availability
- For clinical datasets or third party data, please ensure that the statement adheres to our policy

Source data are provided with this paper. The raw single-cell RNA-seq data generated in this study have been deposited in the NCBI SRA database under the BioProject ID PRJNA994082, available at https://www.ncbi.nlm.nih.gov/bioproject/PRJNA994082. The processed Seurat objects are available at https://doi.org/10.5281/zenodo.14246232

Research involving human participants, their data, or biological material

Policy information about studies with human participants or human data. See also policy information about sex, gender (identity/presentation), and sexual orientation and race, ethnicity and racism.

Reporting on sex and gender	N/A
Reporting on race, ethnicity, or other socially relevant groupings	N/A
Population characteristics	N/A
Recruitment	N/A
Ethics oversight	N/A

Note that full information on the approval of the study protocol must also be provided in the manuscript.

Field-specific reporting

Please select the one below that is the best fit for your research. If you are not sure, read the appropriate sections before making your selection.

☒ Life sciences ☐ Behavioural & social sciences ☐ Ecological, evolutionary & environmental sciences

For a reference copy of the document with all sections, see nature.com/documents/nr-reporting-summary-flat.pdf

Life sciences study design

All studies must disclose on these points even when the disclosure is negative.

Sample size	A power analysis was performed to determine the number of animals in groups at 80% power and type 1 error of 5% based on standard deviations from our previous studies (Nat Commun. 2023 Nov 29;14(1):7852, Sci Immunol. 2020 Apr 10;5(46):eaaz4415, J Exp Med. 2018 Jan 2;215(1):357-375) and on requirements for determining statistical significance. Sample sizes were reported in each figure legend.
Data exclusions	No data was excluded from the analyses.
Replication	All data were obtained from at least two independent experiments. The number of replication is indicated in each figure legend.
Randomization	Experimental groups were randomized across cages and litters, and the location of mice cages was randomized on the racks.
Blinding	Experiments were processed without blinding to ensure accurate group definitions (naive vs uninfected or WT vs knockout), but the analysis was performed with blinding.

Reporting for specific materials, systems and methods

We require information from authors about some types of materials, experimental systems and methods used in many studies. Here, indicate whether each material, system or method listed is relevant to your study. If you are not sure if a list item applies to your research, read the appropriate section before selecting a response.

Materials & experimental systems

n/a	Involved in the study
☐	☒ Antibodies
☒	☐ Eukaryotic cell lines
☒	☐ Palaeontology and archaeology
☐	☒ Animals and other organisms
☒	☐ Clinical data
☒	☐ Dual use research of concern
☒	☐ Plants

Methods

n/a	Involved in the study
☒	☐ ChIP-seq
☐	☒ Flow cytometry
☒	☐ MRI-based neuroimaging

Antibodies

Antibodies used

BD Horizon™ BUV395 Mouse Anti-Mouse CD45.2 - clone 104 (RUO) / cat# 564616 / BD Biosciences
BD OptiBuild™ BUV805 Mouse Anti-Mouse CD45.1 - clone A20 (RUO) / cat# 741958 / BD Biosciences
Clone A20 (RUO)APC/Cyanine7 anti-mouse F4/80 Antibody - clone BM8 / cat# 123118 / Biolegend
Brilliant Violet 421™ anti-mouse F4/80 Antibody - clone BM8 / cat# 123132 / Biolegend
Brilliant Violet 605™ anti-mouse/human CD11b Antibody - clone M1/70 / cat# 101257 / Biolegend
Brilliant Violet 510™ anti-mouse Ly-6C Antibody - clone HK1.4 / cat# 128033 / Biolegend
Alexa Fluor® 700 anti-mouse Ly-6G Antibody - clone 1A8 / cat# 127622 / Biolegend
Alexa Fluor® 488 anti-mouse Ly-6G Antibody - clone 1A8 / cat# 127626 / Biolegend
Brilliant Violet 421™ anti-mouse CD170 (Siglec-F) Antibody - clone S17007L / cat# 155509 / Biolegend
PerCP anti-mouse I-A/I-E Antibody - clone M5/114.15.2 / cat# 107624 / Biolegend
PE anti-mouse Tim-4 Antibody - clone RMT4-54 / cat# 130006 / Biolegend
PE anti-mouse CD138 (Syndecan-1) Antibody - clone 281-2 / cat# 142504 / Biolegend
Alexa Fluor® 647 anti-mouse CLEC4F Antibody - clone 3E3F9 / cat# 156804 / Biolegend
PE/Cyanine7 anti-mouse CD64 (FcγRI) Antibody - clone X54-5/7.1 / cat# 139314 / Biolegend
Brilliant Violet 711™ anti-mouse CD64 (FcγRI) Antibody - clone X54-5/7.1 / cat# 139311 / Biolegend
Alexa Fluor® 647 anti-mouse CD31 Antibody - clone MEC13.3 / cat# 102516
TIM-4 Monoclonal Antibody, PerCP-eFluor™ 710 - clone 54 (RMT4-54) / cat# 46-5866-82 / ThermoFisher, eBioscience
iNOS Monoclonal Antibody Alexa Fluor™ 488 - clone CXNFT / cat# 53-5920-82 / ThermoFisher, eBioscience
F4/80 Monoclonal Antibody Alexa Fluor™ 532 - clone BM8 / cat# 58-4801-82 / ThermoFisher, eBioscience
TIM-4 Monoclonal Antibody, Alexa Fluor™ 488 - clone 54 (RMT4-54) / cat# 53-5866-82 / ThermoFisher, eBioscience
Cleaved Caspase-3 (Asp175) Rabbit mAb #9661 - polyclonal / cat# 9661S / Cell Signalling Technology
Ki-67 (D3B5) Rabbit mAb #9129 - clone D3B5 / cat# 9129S / Cell Signalling Technology
Anti-rabbit IgG (H+L), F(ab')2 Fragment (Alexa Fluor® 488 Conjugate) - cat# 4412S / Cell Signalling Technology
Anti-leishmania antibody - produced in house (described in methods)

Validation

All antibodies from commercial sources have been validated by the vendors, as follows: as follows; 1) Testing on multiple cell and tissue types with a variety of known expression levels. 2) Validation in multiple applications as a cross-check for specificity and to provide additional clarity for researchers. 3) Comparison to existing antibody clones. 4) Using cell treatments to modulate target expression, such as phosphatase treatment to ensure phospho-antibody specificity. The details of validation methods and materials are also available on respective manufacturer home pages for each antibody. BD Biosciences: https://www.bdbiosciences.com/en-us/products/reagents/flow-cytometry-reagents/research-reagents/quality-and-reproducibility
Biolegend: https://www.biolegend.com/ja-jp/quality/quality-control
ThermoFisher, eBioscience: https://www.thermofisher.com/us/en/home/life-science/antibodies/invitrogen-antibody-validation.html
Cell Signalling - https://www.cellsignal.com/about-us/cst-antibody-validation-principles
The anti-leishmania antibody have been validated in our laboratory using samples infected with different Leishmania species and uninfected samples, and testing multiple dilutions.

Animals and other research organisms

Policy information about studies involving animals; ARRIVE guidelines recommended for reporting animal research, and Sex and Gender in Research

Laboratory animals

C57BL/6NTac (Taconic), B6 CD45.1 Jackson line #002014 (Taconic #8478), Ccr2-/- Jackson line #004999 (Taconic #8456), Caspase1-/- (Taconic #8460), Clec4f-Cre-tdTomato (JAX stock #033296), Ai6(RCL-ZsGreen) (JAX stock #007906), R26R-Confetti (JAX stock #013731), Bach1-/-, Mlkl-/- (JAX stock #037116) mice between 6-8 weeks old were used in the study. Colonies were maintained under specific pathogen-free conditions, at a constant cycle of 14 hours in the light (<300 lux) and 10 hours in the dark, at 20–24°C and 40–60% humidity, with free access to food and water.

Wild animals

The study did not involved wild animals

Reporting on sex

Mice used in the study were females that were selected based on our previous publications (Nat Commun. 2023 Nov 29;14(1):7852, Sci Immunol. 2020 Apr 10;5(46):eaaz4415, J Exp Med. 2018 Jan 2;215(1):357-375).

Field-collected samples

This study did not involve samples collected from the field.

Ethics oversight	The experiments were approved by the NIAID Animal Care and Use Committee (protocol number LPD 68E).

Note that full information on the approval of the study protocol must also be provided in the manuscript.

Plants

Seed stocks	N/A
Novel plant genotypes	N/A
Authentication	N/A

Flow Cytometry

Plots

Confirm that:

☐ The axis labels state the marker and fluorochrome used (e.g. CD4-FITC).

☒ The axis scales are clearly visible. Include numbers along axes only for bottom left plot of group (a 'group' is an analysis of identical markers).

☒ All plots are contour plots with outliers or pseudocolor plots.

☒ A numerical value for number of cells or percentage (with statistics) is provided.

Methodology

Sample preparation	Mice were euthanized in a CO2 chamber and immediately perfused with 20 mL sterile 1x PBS. The gallbladder was removed, and the livers were weighed and maintained in 1x PBS at 4°C until processing. Livers were place in 3mL of a digestion solution containing 0.5% collagenase IV and 0.5 mg/mL DNAse in RPMI medium and incubated for 30 minutes at 37°C. The digested livers were filtered through a 70Um cell strainer using 1x PBS. For leukocyte purification, cells were centrifuged in a 34% Percoll solution, prepared in 1x PBS, at 500xg for 20 minutes at 25°C. Red blood cell were lysed using ACK lysis buffer, and the remaining cells were washed once with 1x PBS.
Instrument	Cytek® Aurora cytometer was used for flow cytometry and Cytek® Aurora CS for sorting.
Software	SpectroFlo (version 3.0.1 to 3.3.0) and FlowJo (version 10.8.1 to 10.10.0).
Cell population abundance	The purity of sorted samples was determined by acquiring a small fraction of one of the sorted samples after sorting. The purity for the sorted F4/80hiCD11bintCD64+ from an uninfected mouse was 94% of the CD45+ cells. The purity for the sorted F4/80hiCD11bintCD64+ from an infected mouse was 75% of the CD45+ cells. The purity for the sorted F4/80hiCD11bintCD64+CLEC4F-TIM-4+ from an infected mouse was 78% of the CD45+ cells.
Gating strategy	Gating was first based on FSC/SSC and FSC-H/FSC-A for single cells. Dead cells were excluded by Live/Dead Fixable Blue staining and myeloid populations were gated as: F4/80hiCD11bintCD64+CLEC4F+TIM-4+ (Kupffer cells), F4/80hiCD11bintCD64+CLEC4F+TIM-4- (moKCs), F4/80hiCD11bintCD64+CLEC4F-TIM-4+ (resKC-derived macrophages), F4/80hiCD11bintCD64+CLEC4F-TIM-4- (momacs), CD11b+Ly6G+SiglecF- (neutrophils), CD11b+Ly6G-SiglecF+ (eosinophils) and CD11b+Ly6G-SiglecF-Ly6C+CD64+ (monocytes), CD11b+Ly6G-SiglecF-Ly6C-CD64+MHCII+ (mo-cells).

☒ Tick this box to confirm that a figure exemplifying the gating strategy is provided in the Supplementary Information.
